# Quantization of geometric phase with integer and fractional topological characterization in a quantum Ising chain with long-range interaction

**DOI:** 10.1038/s41598-018-24136-1

**Published:** 2018-04-12

**Authors:** Sujit Sarkar

**Affiliations:** Poornaprajna Institute of Scientific Research, 4 Sadashivanagar, Bangalore, 560 080 India

## Abstract

An attempt is made to study and understand the behavior of quantization of geometric phase of a quantum Ising chain with long range interaction. We show the existence of integer and fractional topological characterization for this model Hamiltonian with different quantization condition and also the different quantized value of geometric phase. The quantum critical lines behave differently from the perspective of topological characterization. The results of duality and its relation to the topological quantization is presented here. The symmetry study for this model Hamiltonian is also presented. Our results indicate that the Zak phase is not the proper physical parameter to describe the topological characterization of system with long range interaction. We also present quite a few exact solutions with physical explanation. Finally we present the relation between duality, symmetry and topological characterization. Our work provides a new perspective on topological quantization.

## Introduction

The physics of Berry Phase (geometric phase) has been playing an important role in understanding pivotal findings of quantum condensed-matter systems^1–22^. The concept of geometric phase was put first by Pancharatnam^23^ in 1956, in the context of interference of polarized light. However the generalization of Berry’s concept has been carried out by Wilczek and Zee^24^ and also by Aharonov and Anandan^25^ independently. The motivation of this research presented here is derived from the seminal work of Berry. Berry has found this phase along with the dynamical phase of the system for the cyclic evolution of a wave function and, at the same time, this phase is gauge invariant. Here, we mention very briefly the basic aspect of geometric phase to emphasis the importance of the present study of quantization of geometric phase from the perspective of integer and fractionally topological characterization. One finds the time evolution of the Hamiltonian through the time dependent Schrödinger equation

$$H(R(t))|{\psi }(t) > =ih{\partial }_{t}|{\psi }(t) > \,.$$

The Hamiltonian of the system depends on the parameter *R*(*t*) which changes with time adiabatically. One can also write this equation in a basis |*ϕ*(*x*(*t*)) > corresponding to the energies *E*_*n*_ as, *H*(*R*(*t*))|*ϕ*(*R*(*t*)) > = *E*_*n*_(*R*(*t*))|*ϕ*(*R*(*t*)) >.

Berry assumes that the properties of the Hamiltonian is such that there is no degeneracy and no level crossing in the system during the evolution. During the adiabatic time evolution of the system, the state vector acquires an extra phase over the dynamical phase, $|{\psi }(R(t)) > ={e}^{{\alpha }_{n}}|{\varphi }(R(t) >$, where *α*_*n*_ = *θ*_*n*_ + *γ*_*n*_. ${\theta }_{n}(=\frac{-1}{h}{\int }_{0}^{t}{E}_{n}(\tau )d\tau )$ and *γ*_*n*_ are the dynamical and geometric phases respectively. Finally, Berry obtained the geometric phase as

$${\gamma }_{n}(C)=i{\int }_{C} < {\varphi }(x)|\nabla |{\varphi }(x) > dx,$$

when the system is given to the cyclic evolution described by a closed curve. It is evident from the analytical expression that Berry phase depends on the geometry of the parameter and loop (C) therein. This is the basic origin of geometrical character of the Berry phase.

Here, we mention very briefly the famous example of geometric phase of spin-1/2 particle, which is moving in an external magnetic field $\overrightarrow{B}$ rotating adiabatically under an angle *θ* around z-axis. The time dependent external magnetic field can be written as, *B*_*x*_(*t*) = *B*_0_ sin *θ* cos (*ωt*), *B*_*y*_(*t*) = *B*_0_ sin *θ* sin (*ωt*), *B*_*z*_(*t*) = *B*_0_ cos *θ*, where *ω* is the angular frequency of the rotation and *B*_0_ = |$\overrightarrow{B}$(*t*)|. The Berry phase can be evaluated very easily as

1 $$\begin{array}{rcl}{{\gamma }}_{\pm } & = & i{\int }_{C} < {{\psi }}_{\pm }|\nabla |{\psi }_{\pm } > {B}_{0}\,\sin \,\theta d{\varphi },\\  & = & -{\pi }(1\mp \,\cos \,\theta ),\end{array}$$

where |*ψ*_±_ > are the eigenstates with energies *E*_±_ = ±*μB*_0_. The curve *C* in the parameter space is a sphere due to the existence of three component of an external magnetic field, *B*_0_ = constant, *θ* = constant, *ε* ∈ [0, 2*π*]. One can write the above equation as half of the solid angle enclosed by the path *C*,

2 $${{\gamma }}_{\pm }(C)=\mp \frac{1}{2}{\rm{\Omega }}(C).$$

We will use this result of *γ*_±_(*C*) for the physical arguments of topological nature of geometric phase in the following sections of this work.

The Berry phase is geometric in nature through the dependence of local geometry of the path (*C*), i.e., the geometric phase will change if the path changes. However, there are some situations when the Berry phase remains the same, that is when the path (*C*) is subjected to smooth transformation, for this situation the geometric phase is topological in nature. The most fundamental example to illustrate this concept is Aharonov-Bohm effect (${\gamma }_{n}(C)=\frac{e}{\bar{h}}{\int }_{C}A\mathrm{.}\,dR=\frac{e{\rm{\Phi }}}{\hslash }$, where *γ*_*n*_(*C*) is the geometric phase. It is independent of *n* and *C*, here the parametric space is $({{\mathbb{R}}}^{2})$ whereas the parametric space of spin-1/2 particle moving in a external magnetic field is (${\mathbb{R}}3$). Therefore, the solid angle appears for this situations, however there is no scope of solid angle for the Aharnov-Bohm effect because the parameter space is (${{\mathbb{R}}}^{2}$). It is entirely topological in origin. The whole physical explanation become clear after the seminal work of Berry^1^). The topological phase is non-local in the sense that it cannot be defined at a point in space, but only as a closed integral enclosing the magnetic flux or what is observed in the Aharonov-Bohm effect^26^. In the latter phase, Aharonov finds another effect (topological phase) which goes by the name “Aharonov-Casher” effect^27^. The problem which we solve here is topological in nature with many new and interesting features and has not been explored explicitly in the literature of this model Hamiltonian and its variant system.

Now, we state very briefly why the topological quantum phase transition is so interesting and important?

While the conventional quantum phases are described by continuous order parameters^28,29^, the topological ones are characterized by quantized topological invariants, which correspond to topological properties of occupied quantum state. Landau’s theory of phase transition is related to the local order parameter, but in quantum many body condensed matter system, there are several examples of topological order which do not have a local order parameter. However, there is no Landau like theory to describe the topological state of matter. These topological properties are robust under small adiabatic deformations of the Hamiltonian, and changing them requires so-called topological phase transitions, which do not accompany symmetry breaking in contrast to conventional quantum phase transitions. But are symbolized by gap closing at some specific points in the Brillouin zone (BZ). However, topological invariants are ill-defined at topological phase transition points, as they are usually defined for each quantum state protected by non-zero energy gaps.

Quantum spin models have got considerable attraction in the condensed matter physics community for the following reasons. Firstly, the quantum spin model can be simulated in an artificial quantum system with tunable parameters. Secondly, quantum simulations of the spin chain systems can be realized through different physical systems^30,31^. Furthermore, these systems are test-beds for applying new ideas and methods to quantum phase transition. Topological properties of quantum matter and interacting light matter systems are also in the state of art to understand many basic and fundamental aspects of topological states of matter^32–41^.

## This research paper has some goals

C. N. Yang, in his concluding talk of the TH 2002 conference in Paris characterized the twentieth century theoretical physics by three “Melodies”. “Symmetry, quantization and phase factor^42^. This comment of the legendary theoretical physicist motivates us to study the quantization of geometrical phase, duality and symmetry of this model Hamiltonian system. The detailed motivation of this research paper is presented below.

In this study, the quantum Ising model with long range interaction is considered. The change in the topological characterization due to the introduction of the next-nearest-neighbor interaction in the model Hamiltonian is attempted to be elucidated. For this situation, three quantum critical lines are obtained (please see the “Method” section for detail derivation for the quantum critical lines).

A pertinent question arises: Are the all quantum critical lines the same in nature from the perspective of topological characterization? This problem is studied explicitly for this model Hamiltonian.

A one-dimensional topological system is characterized by the topological invariant number (winding number). The system is in the non-topological state for the zero winding number with the absence of zero mode Majorana alike excitation at the edge of the system. For the system with the integer winding number is in the topological state with the integer numbers of zero energy Majorana edge mode at both the edges of the system^43–63^. This is the bulk boundary correspondence. The common notion is that bulk boundary remains unchanged for the Hermitian system. The system with non-Hermiticity shows the physics beyond the bulk boundary correspondence.

The authors of refs^64,65^, have shown explicitly that the bulk boundary correspondence can be modified in the presence of non-Hermiticity. They have shown that for a non-Hermitian Hamiltonian that encircles an exceptional point in the momentum space, the winding number has a fractional value 1/2. In this study, model Hamiltonian is Hermitian. However, any possibility to find the physics beyond the bulk boundary correspondence is explored. The possibility to find the fractional values of winding numbers for this study even though the model Hamiltonian has no exceptional point in the momentum space has been explored in this study.

We search what is the relation between the duality transformation and the topological quantization. Apart from that, quite a few exact solutions for the quantization of geometric phase with a physical explanation are presented.

We also do the study for the symmetry operations for this model Hamiltonian explicitly to ascertain any difference of symmetries for the integer and the fractional topological characterization of the system.

The other most important part of this study is how the topology of the auxiliary space changes for the integer and fractional topological characterization, along with the physical interpretation of integer and fractional topological characterization.

Based on our results on quantization of Zak phase, we would like to raise the question whether the Zak phase is at all meaningful for the topological characterization of the system with long range interaction. There are quite a few studies in the literature of quantum condensed matter physics for studying the topological state and the properties of system through the Zak phase. However, the behavior of Zak phase under the integer and fractional topological characterization is absent in the literature^1–22^.

The quantum phase transition properties have already been studied in quantum Ising model. At the same time, Ising models with long range interaction have been found to exhibit topological characterization, but the study of quantization of geometric phase with integer and fractional topological characterization for this quantum Ising model with long range interaction is absent in the literature^66–70^.

This work provides a new perspective on topological quantization.

## Model Hamiltonian and geometric phase calculations

The model Hamiltonian^68^ of the present study is

3 $$H=-\,\sum _{i}({\mu }{{\sigma }}_{i}^{x}+{{\lambda }}_{2}{{\sigma }}_{i}^{x}{{\sigma }}_{i-1}^{z}{{\sigma }}_{i+1}^{z}+{{\lambda }}_{1}{{\sigma }}_{i}^{z}{{\sigma }}_{i+1}^{z}).$$

It is clear from the above Hamiltonian that the transverse field Ising model is modified by the presence of next-nearest-neighbor spins interaction. *λ*_1_ and *λ*_2_ are the nearest-neighbor (NN) and next-nearest-neighbor (NNN) interactions respectively and *μ* is the strength of the transverse coupling. Here we consider it as a chemical potential.

This Hamiltonian transfer to the spinless fermion Hamiltonian through the Jordan-Wigner transformation, ${{\sigma }}_{i}^{x}=(1-2{{c}_{i}}^{\dagger }{c}_{i})$, ${{\sigma }_{i}}^{z}={{\rm{\Pi }}}_{j < i}(1-2{{c}_{j}}^{\dagger }{c}_{j})({c}_{j}+{{c}_{j}}^{\dagger })$.

After the Jordan-Wigner transformation, the Hamiltonian is reduced to,

4 $$H=-\,{\mu }\sum _{i=1}^{N}(1-2{{c}_{i}}^{\dagger }{c}_{i})-{{\lambda }}_{1}\sum _{i=1}^{N-1}({{c}_{i}}^{\dagger }{c}_{i+1}+{{c}_{i}}^{\dagger }{{c}_{i+1}}^{\dagger }+h.\,c)-{{\lambda }}_{2}\sum _{i=1}^{N-1}({{c}_{i-1}}^{\dagger }{c}_{i+1}+{c}_{i+1}{c}_{i-1}+h.\,c).$$

Finally, after the Fourier transform, the Hamiltonian become,

5 $$\begin{array}{rcl}H & = & \sum _{k}\mathrm{(2}{\mu }-2{{\lambda }}_{1}\,\cos \,k-2{{\lambda }}_{2}\,\cos \,2k){{c}_{k}}^{\dagger }{c}_{k}\\  &  & +i\sum _{k}\mathrm{(2}{{\lambda }}_{1}\,\sin \,k{{c}_{k}}^{\dagger }{{c}_{-k}}^{\dagger }+2{{\lambda }}_{2}\,\sin \,2k{{c}_{k}}^{\dagger }{{c}_{-k}}^{\dagger }+H\mathrm{.}C\mathrm{).}\end{array}$$

Now we are interested to derive the analytical expression of geometric (Zak) phase. Basic definition of Zak phase is the following. The Berry’s phase picked up by a particle moving across the Brillouin zone. Here, the Brillouin zone is in the one dimension as treated by the Zak, and, therefore, the natural choice for the cyclic parameter is the crystal momentum (*k*). The geometric phase in the momentum space is defined as

6 $${{\gamma }}_{n}={\int }_{-\pi }^{\pi }dk < {u}_{n,k}|i{\partial }_{k}|{u}_{n,k} > \,,$$

where |*u*_*n*,*k*_ > is the Bloch states which are the eigenstates of the *n*^*th*^ band of the Hamiltonian. The model Hamiltonian of the present problem is *Z* type topological invariant and the system has an anti-unitary particle hole symmetry (please see the “Symmetry” section for the detailed symmetry operations). For this system, the analytical expressions of the Zak phase^3,14,19^ is

7 $${\gamma }=W\pi \,\,{\rm{mod}}\,\mathrm{(2}\pi \mathrm{).}$$

Now the main task is to calculate the topological invariant (winding number).

The BdG equation for this Hamiltonian is

8 $${H}_{BdG}=(\begin{array}{cc}{\chi }_{z}(k) & i{\chi }_{y}(k)\\ -i{\chi }_{y}(k) & -{\chi }_{z}(k)\end{array})$$

where,

9 $${{\chi }}_{y}(k)=2{{\lambda }}_{1}\,\sin \,k+2{{\lambda }}_{2}\,\sin \,2k,$$

10 $${{\chi }}_{z}(k)=-\,2{{\lambda }}_{1}\,\cos \,k-2{{\lambda }}_{2}\,\cos \,2k+2{\mu },$$

11 $${E}_{(k)}=\sqrt{{{\chi }}_{z}{(k)}^{2}+{{\chi }}_{y}{(k)}^{2}}\mathrm{.}$$

Topological phase transition can be ascribed by the topological invariant quantity. It is convenient to define this invariant quantity using the Anderson pseudo-spin approach^71^. One can write BdG Hamiltonian in the pseudo spin as,

12 $$\vec{{\chi }}(k)={{\chi }}_{y}(k)\vec{y}+({{\chi }}_{z})(k)\vec{z}\mathrm{.}$$

13 $${H}_{BdG}(k)=\sum _{i}\vec{{\chi }}{(k)}^{i}\mathrm{.}{\vec{{\tau }}}^{i},$$

where *τ*’s are the Pauli matrices which act in the Nambau basis of *H*_*BdG*_. Finally, we succeeded in presenting the model Hamiltonian as a pseudo spin in a magnetic field in a two-dimensional plane (YZ plane). The parameter space of the Hamiltonian is (${{\mathbb{R}}}^{2}$) but this space is locally flat, whereas the parameter space for a spin-1/2 electron in a rotating magnetic field is sphere (${{\mathbb{S}}}^{2}$), which is obviously a curve (Equation 1). We notice from the example of spin-1/2 particle in a rotating magnetic field that the geometric phase is the half of the solid angle (Equation 2). For the present problem, there is no opportunity for the solid angle for the following reasons: The model Hamiltonian has only two components of magnetic field and therefore the parametric space is (${{\mathbb{R}}}^{2}$), i.e., the parameter space is flat, as a consequence of it there is no scope for the appearance of solid angle as we discuss in the introduction for Aharnov-Bohm effect. This physical explanation of topological characterization is consistent with the discussions of ref.^72^.

One can write the vector $\hat{{\chi }}(k)$ as,

14 $$\hat{{\chi }}(k)=\,\cos \,{{\theta }}_{k}\hat{y}+\,\sin \,{{\theta }}_{k}\hat{z}\mathrm{.}$$

and $${{\theta }}_{k}={ta}{{n}}^{-1}(\frac{{\chi }_{y}(k)}{{\chi }_{z}(k)})$$.

15 $$W=\frac{1}{2\pi }\int \frac{d\theta (k)}{dk}dk\mathrm{.}$$

16 $${\gamma }=\frac{1}{2}\int \frac{d\theta (k)}{dk}dk\,{\rm{mod}}\,\mathrm{(2}{\pi }\mathrm{).}$$

The winding number (*W*) presents the number of times $\hat{{\chi }}(k)$ rotates in the YZ plane around the Brillouin zone. It reveals from the above mentioned equation that the variation of $\frac{d\theta (k)}{dk}$ across the Brillouin zone boundary carries the most important feature for the nature of geometric phase.

The general expression for the geometric phase (Zak phase) for this model Hamiltonian is

17 $${\gamma }={\int }_{-\pi }^{\pi }\frac{{{\lambda }}_{1}\mathrm{(6}{{\lambda }}_{2}-2{\mu })\,\cos \,k+\mathrm{2(}{{{\lambda }}_{1}}^{2}+2{{{\lambda }}_{2}}^{2}-2{\mu }{{\lambda }}_{2}\,\cos \,2k)}{\mathrm{(4}{{{\lambda }}_{1}}^{2}+4{{{\lambda }}_{2}}^{2}+4{{\mu }}^{2}+4{{\lambda }}_{1}\mathrm{(2}{{\lambda }}_{2}-2{\mu })\,\cos \,k-8{\mu }{{\lambda }}_{2}\,\cos \,2k)}\,dk\,{\rm{mod}}\,\mathrm{(2}{\pi }\mathrm{).}$$

Justification of the derivation of above equation is the following: The authors of refs^14,19^ have shown explicitly from the symmetry arguments of the model Hamiltonian that the Zak phase is related with the winding number (*W*) by the Eq. 7. The symmetry of our model Hamiltonian is the same as that of refs^14,19^. We calculate winding number by using Eq. 15 (P. W. Anderson pseudo spin approach ref.^71^). Finally we obtain the general expression for geometric phase (Zak phase). This is the justification for the derivation of the geometric phase (Zak Phase).

## Results

Here, we study the quantization of geometric phase in the different regime of the parameter space of the system, which are, (1) *λ*_2_ = 0 and *λ*_1_ ≠ 0, (2) *λ*_1_ = 0 and *λ*_2_ ≠ 0, (3) *λ*_2_ = −*μ*. (4) *λ*_2_ = *μ* + *λ*_1_, (5) *λ*_2_ = *μ* − *λ*_1_, The last three lines are the quantum critical lines.

### Quantization of geometric phase with integer topological characterization

In this section, we discuss the results regarding Zak phase and their physical consequences with integer topological characterization, i.e., winding number takes the integer values.

Before we present the results, we would like to state a few generic information about the presentation. The Zak phase takes only two quantized values 0 and *π* because it is measured in modulo of 2*π*. Therefore, when the winding number is even integer multiple, then the geometric phase is zero because it is measured in the modulo of 2*π*. However in the figure, we present the geometric phase, *γ* as 0,*π* and 2*π*, to state the different topological state of the system in spite of the geometric phase remaining the same with *γ* = 0 and *γ* = *π* for even and odd integer multiple of winding number respectively.

In our study, we observe a region of parameter space, where the system shows the integer topological characterization. This parameter space is stated as: (1) *λ*_1_ ≠ 0, *λ*_2_ = 0, (2) *λ*_2_ ≠ 0, *λ*_1_ = 0, (3) *λ*_2_ = −*μ*. The analytical expressions for the geometric phase for these regime of the parameter space are the following

18 $${\gamma }^{\mathrm{(1)}}={\int }_{-\pi }^{\pi }\frac{-2{\lambda }_{1}\mu \,\cos \,k+2{{\lambda }_{1}}^{2}}{\mathrm{(4}{{\lambda }_{1}}^{2}+4{\mu }^{2}-8{\lambda }_{1}\mu \,\cos \,k)}\,dk\,{\rm{mod}}\,\mathrm{(2}\pi ),$$

where *λ*_1_ ≠ 0 but *λ*_2_ = 0.

19 $${\gamma }^{\mathrm{(2)}}=2{\int }_{-\pi }^{\pi }\frac{-2{\lambda }_{2}\mu \,\cos \,2k+2{{\lambda }_{2}}^{2}}{\mathrm{(4}{{\lambda }_{2}}^{2}+4{\mu }^{2}-8{\lambda }_{2}\mu \,\cos \,2k)}\,dk\,{\rm{mod}}\,\mathrm{(2}\pi ),$$

where *λ*_2_ ≠ 0 but *λ*_1_ = 0.

20 $${\gamma }^{\mathrm{(3)}}={\int }_{-\pi }^{\pi }\frac{-\mathrm{8\ }{\lambda }_{1}\mu \,\cos \,k+\mathrm{2(}{{\lambda }_{1}}^{2}+2{\mu }^{2}+2{\mu }^{2}\,\cos \,2k)}{\mathrm{(4}{{\lambda }_{1}}^{2}+8{\mu }^{2}-16{\lambda }_{1}\,\mu \,\cos \,k+8{\mu }^{2}\,\cos \,2k)}\,dk\,{\rm{mod}}\,\mathrm{(2}\pi ),$$

where *λ*_2_ =−*μ*. The analytical expressions for Zak phase are ill define at the topological quantum phase transition points because the winding number is ill define at that points due to the change of topological state at that point.

**Figure 1:**
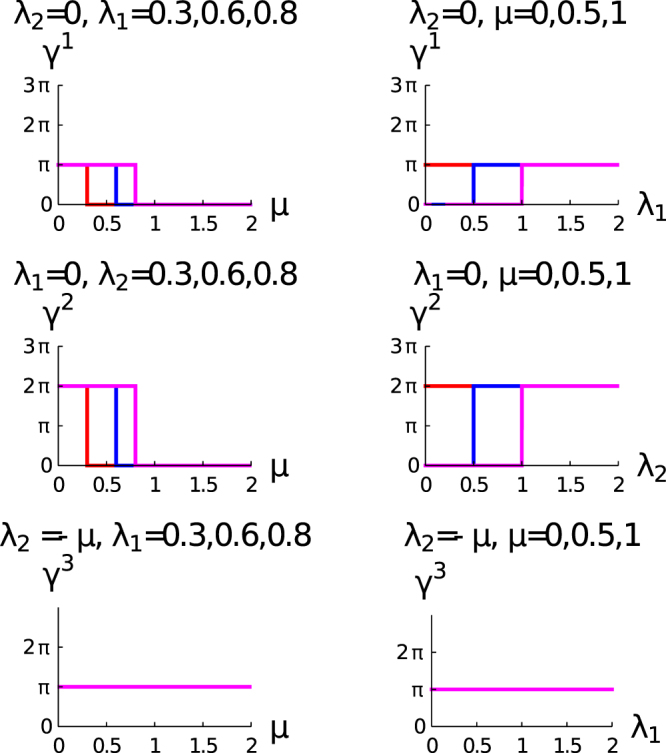
The left and the right columns show respectively the variation of geometric phase (*γ*) with *μ* and *λ*_1_. *λ*_1_ ≠ 0 and *λ*_2_ = 0, *λ*_2_ ≠ 0 and *λ*_1_ = 0 and *λ*_2_ =−*μ* are for upper, middle and lower row respectively. The curves in the right column of this figure for *μ* = 0, 0.5, and 1 are for red, blue and magenta respectively. The curves in the left column for the first and third row of this figure for *λ*_1_ = 0.3,0.6 and 0.8 are for red, blue and magenta respectively. The curves in the left column for the second row of this figure for *λ*_2_ = 0.3,0.6 and 0.8 are red, blue and magenta respectively.

Figure 1 consists of three rows: the upper, middle and lower are *λ*_1_ ≠ 0 and *λ*_2_ = 0; *λ*_2_ ≠ 0 and *λ*_1_ = 0; *λ*_2_ =−*μ* respectively. Our study reveals that the *γ*^(1)^ is non-zero when *λ*_1_ > *μ*, otherwise it is zero, i.e., the transition occurs at the *λ*_1_ = *μ*. The variation of *γ*^(1)^ with *λ*_1_ reveals that the transition of *γ*^(1)^ from zero to *π* occurs at *λ*_1_ = *μ*, i.e., from the topologically trivial state to the topological state. We observe only a single transition which is related to the topological state of the system with a change of winding number unity, i.e., the system is in the state with integer topological characterization. Therefore, in this regime of parameter space the topological characterization of the state is the same both for Zak phase and winding number study. Only at the topological quantum phase transition point the jump of the winding number is sharp and it is ill define otherwise winding number is definite for the topological state. One can also explain this situation physically: The topological invariant quantity depends on the topology of the configuration space, for a particular topological configuration space winding number is a definite value, and change of winding number leads the system to the different topological configuration. This change of configuration space never become continuous because it is topological quantum phase transition, and not the continuous phase transition with order parameter. This is the physical explanation of sharp change of winding number at the topological quantum phase transition point.

The results and physical analysis of our study are consistent with the previous studies^14,73^, they have done only for the short range interaction.

**Figure 2:**
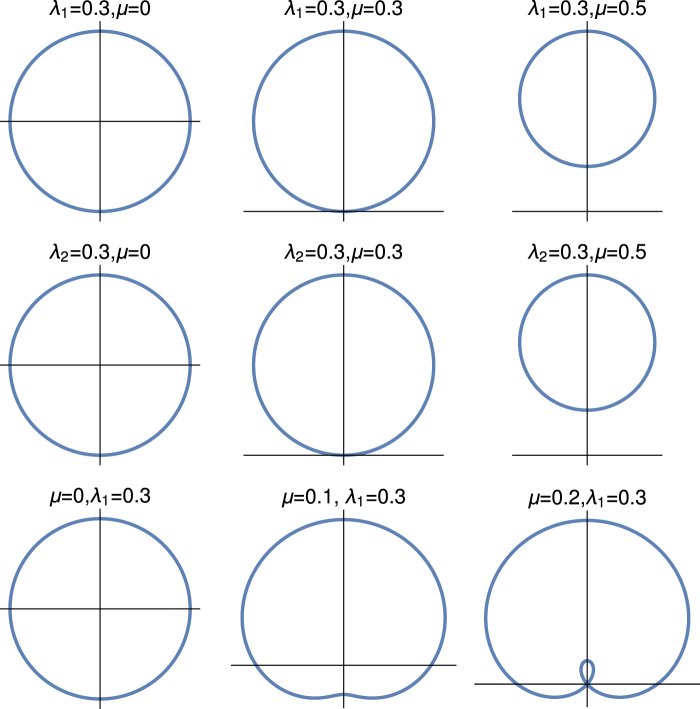
Parametric plot of the vector $\hat{{\chi }}(k)$. The upper, middle and lower rows of this figure are *λ*_1_ ≠ 0, *λ*_2_ = 0; *λ*_2_ ≠ 0 and *λ*_1_ = 0, and *λ*_2_ =−*μ* respectively.

The authors of ref.^63^ have also found the transition of geometric phase from 0 to *π* for the topological orbital ladders. We observe in the middle row, the geometric phase that shows transition from 0 to 2*π* for *λ*_2_ > *μ*. For this transition, the winding number of the system changes from 0 to 2 whereas the geometric phase remains 0, but we present in the figure as 2*π* to illustrate the distinction between the two different topological states of the system and this is also in the integer topological characterization. The third row is always in the topological state with *γ*^(3)^ = *π* and remains constant, i.e., the system is in the topological state with winding number unity, and there is no transition of *γ*^(3)^. Here, the system also shows the integer topological characterization, this description is only valid when the system is in gapped phase otherwise the situation is different. In Fig. 2, we present both the gapped and gapless feature of this quantum critical line explicitly. We also find quite a few exact solutions for different regime of the parameter space which also support these results.

We observe that the geometric phase (Zak phase) only describes the topological characterization correctly if it takes only two values 0 and *π*, like to the Kitaev’s model, where the system is either in the non-topological state (*W* = 0, *γ* = 0) or the topological state with (*W* = 1, *γ* = *π*). However, the system with long range interaction, the Zak phase will not depict the right physical picture because, for the same value of Zak phase, the number Majorana alike zero mode excitations are different at the edge of the system. Therefore, the results of topological invariant number is more fundamental than the results of Zak phase for the characterization of topological state of matter^74–76^. For that reason, we present our results of Zak phase from the perspective of winding number.

The parametric equation of the Hamiltonian in the pseudo spin space is the following form: *χ*(*k*) = (*χ*^*x*^(*k*), *χ*^*y*^(*k*), *χ*^*z*^(*k*)) = (0, 2*λ*_1_ sin *k* + 2*λ*_2_ sin 2*k*, −2*λ*_1_ cos *k*−2*λ*_2_ cos 2*k* + 2*μ*). The parametric equations for the system are

$$y(k)=-\,2{\lambda }_{1}\,\cos \,k-2{\lambda }_{2}\,\cos \,2k+2\mu ,z(k)=2{\lambda }_{1}\,\sin \,k+2{\lambda }_{2}\,\sin \,2k.$$

We have already discussed the topological origin of the geometric phase for this model Hamiltonian. In Fig. 2, we show explicitly how the unit vector $\hat{{\chi }}(k)$ changes in the auxiliary space due to the change of the parameter space of the model Hamiltonian. This figure consists of three rows: the upper, middle and lower rows and are for *λ*_1_ ≠ 0 and *λ*_2_ = 0; *λ*_2_ ≠ 0 and *λ*_1_ = 0; and *λ*_2_ = −*μ* respectively. The parameter space of these three rows are the same with the parameter space of Fig. 1.

We observe in the auxiliary space that when *λ*_1_ > *μ* and *λ*_2_ = 0, the origin of the parametric space is inside the circle otherwise not. The same observation is for when *λ*_2_ > *μ* and *λ*_1_ = 0. These loop only touch the origin at the topological quantum phase transition point. There is no gapless excitation for these two lines except at the topological quantum phase transition point. But the situation is different for the third row (*λ*_2_ = −*μ*). The system is in the integer topological characterization, when it is in the gapped phase (*λ*_1_ > |2*λ*_2_|). At the gapless phase (*λ*_1_ < |2*λ*_2_|) situation is different, as we observe in the last panel of third row, where the system shows the behaviour of fractional topological characterization. As the chemical potential increases the origin of the auxiliary space shifts from the centre to the edge and finally loop touches the origin of the auxialiary space. We present detail study of fractional topological characterization in the next section. The other consistent picture of this figure which reflect in the first column that for *μ* = 0 the behaviour of all three lines are the same as it should be because at this value of chemical potential, the system is in the topological state for all quantum critical lines.

**Figure 3:**
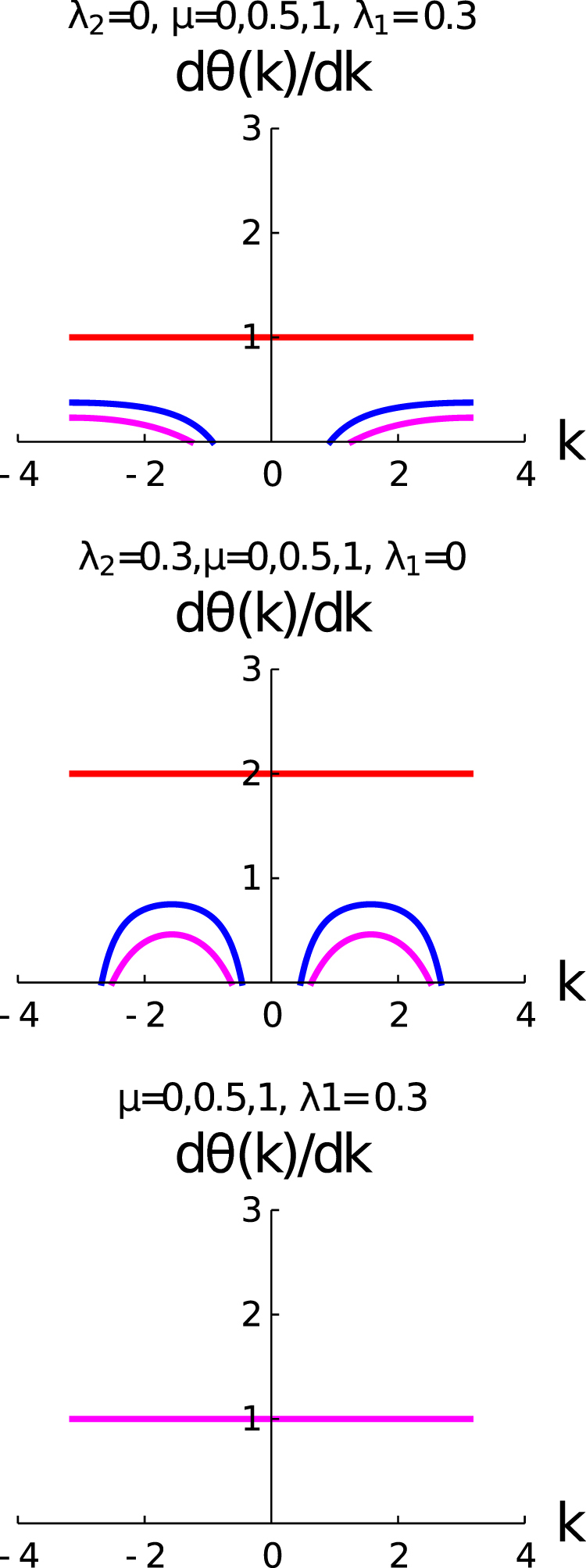
shows the variation of $(\frac{d\theta (k)}{dk})$ with *k*. The upper, middle and lower rows are *λ*_1_ ≠ 0 and *λ*_2_ = 0; *λ*_2_ ≠ 0 and *λ*_1_ = 0, and *λ*_2_ = −*μ* (different colours like red and blue are hiding under the magenta curve) respectively. The curves red, blue and magenta are for *μ* = 0,0.5 and 1 respectively.

Figure 3 consists of three rows: the upper, middle and lower rows are *λ*_1_ ≠ 0 and *λ*_2_ = 0; *λ*_2_ ≠ 0 and *λ*_1_ = 0; and *λ*_2_ = −*μ* respectively. We have already emphasized that the variation of $\frac{d{\theta }_{k}}{dk}$ with *k* plays an important role for the determination of geometric phase of the system. The parameter space of these three rows are the same with the parameter space of the three rows of Fig. 1. In the case of upper row, we observe that for *μ* = 0, there is no variation of $\frac{d{\theta }_{k}}{dk}(\,=\,1)$, which represent the state with *γ* = *π*, whereas the $\frac{d{\theta }_{k}}{dk}$ shows the appreciable variation with k for different values of *μ*s for a fixed value of *λ*_1_. The result reveals from the second row *μ* = 0 that there is no variation of $\frac{d{\theta }_{k}}{dk}(\,=\,2)$ with *k*. It presents the topological state with *γ* = 2*π*(≡0), and $\frac{d{\theta }_{k}}{dk}$ shows the appreciable variation with k for different values of *μ*s for a fixed value of *λ*_2_. The most beautiful feature of this study reveals that $\frac{d{\theta }_{k}}{dk}$ is symmetric with the variation of *k* for this parameter space. In the lower row, $\frac{d{\theta }_{k}}{dk}=1$ for all values of *μ*, and as a consequence of it is *γ*^(3)^ = *π*.

### Quantization of geometric phase with fractional topological characterization

In this section, we present the quantization of geometric phase for the fractionally topological characterization parametric regime of the system, where the winding number takes only the fractional values. We study the quantization of geometric phase for the lines *λ*_2_ = *μ* + *λ*_1_ and *λ*_2_ = *μ* − *λ*_1_. The analytical expression for the geometric phase for these two fractionally topological characterization of the system are *γ*^(4)^(*λ*_2_ = *μ* + *λ*_1_) and *γ*^(5)^(*λ*_2_ = *μ* − *λ*_1_).

21 $${\gamma }^{\mathrm{(4)}}={\int }_{-\pi }^{\pi }\frac{{\lambda }_{1}\mathrm{(6(}\mu +{\lambda }_{1})-2\mu )\,\cos \,k+\mathrm{2(}{{\lambda }_{1}}^{2}-\mathrm{2(}\mu +{\lambda }_{1}{)}^{2}+2\mu (\mu +{\lambda }_{1})\,\cos \,2k)}{\mathrm{(4}{{\lambda }_{1}}^{2}+\mathrm{4(}\mu +{\lambda }_{1}{)}^{2}+4{\mu }^{2}+4{\lambda }_{1}\mathrm{(2(}\mu +{\lambda }_{1})+\mu )\,\cos \,k-8\mu (\mu +{\lambda }_{1})\,\cos \,2k)}\,dk\,{\rm{mod}}\,\mathrm{(2}\pi \mathrm{).}$$

22 $${\gamma }^{\mathrm{(5)}}={\int }_{-\pi }^{\pi }\frac{{\lambda }_{1}\mathrm{(6(}\mu -{\lambda }_{1})-2\mu )\,\cos \,k+\mathrm{2(}{{\lambda }_{1}}^{2}+\mathrm{2(}\mu -{\lambda }_{1}{)}^{2}-2\mu (\mu -{\lambda }_{1})\,\cos \,2k)}{\mathrm{(4}{{\lambda }_{1}}^{2}-\mathrm{4(}\mu -{\lambda }_{1}{)}^{2}+4{\mu }^{2}+4{\lambda }_{1}\mathrm{(2(}\mu -{\lambda }_{1})-2\mu )\,\cos \,k-8\mu (\mu -{\lambda }_{1})\,\cos \,2k)}\,dk\,{\rm{mod}}\,\mathrm{(2}\pi \mathrm{).}$$

**Figure 4:**
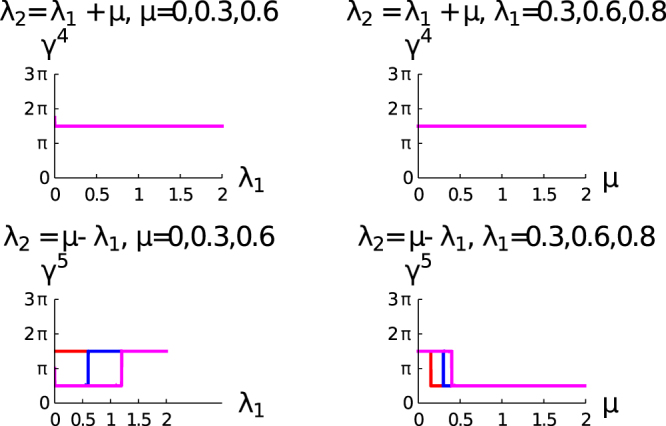
The left and the right column show the variation of geometric phase (*γ*) with *λ*_1_ and *μ* respectively. The upper and lower rows are for *λ*_2_ = *μ* + *λ*_1_ and *λ*_2_ = *μ* − *λ*_1_ respectively. The curves in the right column of this figure are red, blue, and magenta for *μ* = 0,0.5 and 1 respectively. The curves in the left column of this figure are red, blue, and magenta for *λ*_1_ = 0.3,0.6 and 0.8 respectively.

The study of upper panel of Fig. 4 reveals that there is no variation of *γ*^(4)^ with *μ* and *λ*_1_. In the lower panel, where we consider the variation of *γ*^(5)^ for different values of *λ*_1_ and *μ*, we observe that there is a transition from ${\gamma }^{(5)}=\frac{3\pi }{2}$ to $\frac{\pi }{2}$ at $\mu =\frac{{\lambda }_{1}}{2}$. Therefore, we conclude that the geometric phase is also quantized but for a fractional value of *π* and the analytical relation between *λ*_1_ and *μ* are different from the integer topological characterization (*μ* = *λ*_1,2_). The detail derivation for this parametric relation is relegated to the “Method” section. The difference of geometric phase between the two fractionally topological characterization is *π*, which is same with the difference between the trivial and topological state for the integer topological characterization.

Now, we discuss two possible physical picture of the fractional topological characterization. The first physical explanation is the following: The fractionally quantized winding number for the above mentioned two quantum critical lines is not physically realizable; the explanation is the following: For this situation there is no topological excitations in the system. Here one can continuously deform the topological patch to a single point (because the unit vector $\hat{\chi }(k)$ not complete any integer number of rotation), as if the system is in the trivial topological state, with zero value of winding number. This physical situation has explained very nicely in ref.^29^. Therefore, it is very clear from our study that the topological properties of three quantum critical lines are different. Only one of them shows the integer topological characterization for a certain regime of the parameter space. In the “Method” section, we have shown explicitly that for the other two quantum critical lines, the system is always in a gapless phase, apart from the parametric condition of gapless phase. Therefore, due to the presence of gapless state, the system never achieve the gapped topological state. This is main difference between the integer and fractional topological characterization.

The second physical picture is the following: To interpret the fractional topological characterization with gapless edge mode, one can think the redefinition of the “ill-defined” point, i.e., to exclude the gapless points for the quantum critical lines. The band gap vanishes at a specific momentum along a quantum critical line, in order to avoid the ill-definition problem, one can exclude the momentum during the calculation of winding number. In this situation system has no bulk mode, only possesses the edge mode with fractional topological characterization.

There are a few non-Hermitian model Hamiltonian systems with chiral symmetry having an anomalous edge modes^64,65^. These anomalous edge modes are embedded within a complex gapless band structure and have appeared in the vicinity of exceptional points. However, definite clarity whether this physics has any relation to the model independent bulk topological invariant similar to those in Hermitian systems^65^ is yet to be found. This is the main motivation to make a comparison between this study with the study of topological state of non-Hermitian Hamiltonian system.

The model Hamiltonian of the present study is the Hermitian one. However, we have found the presence of fractionally quantized excitation with winding numbers *W* = 3/2, and 1/2. However, the nature of excitation is different from the non-Hermitian system. For the non-Hermitian system, the authors^64,65^ have predicted *W* = 1/2. For their study, there is only one dynamically stable zero-energy edge state due to the non-Hermiticity of the Hamiltonian.

**Figure 5:**
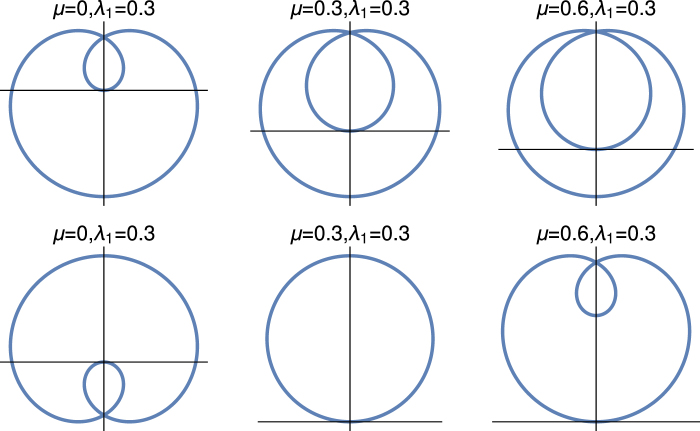
Parametric plot of the $\hat{{\chi }}(k)$. The upper panel is for the *λ*_2_ = *μ* + *λ*_1_ and the lower panel is for *λ*_2_ = *μ* − *λ*_1_.

In Fig. 5, we present the parametric study of $\hat{{\chi }}(k)$ in the auxiliary space for the fractionally topological characterization. The upper and lower rows are for the *λ*_2_ = *μ* + *λ*_1_ and *λ*_2_ = *μ* − *λ*_1_ respectively. Here, we present the results for the same parameter space that has been used in the Fig. 4.

It is very interesting to observe that for fractionally quantized topological characterization, the loop always touch the origin of the auxialary space (*χ*_*y*_ = 0 = *χ*_*z*_). We show explicitly in the “Method” section that for these two quantum critical lines, the system has bulk gapless excitation (at *k* = 0 for *λ*_2_ = *μ* − *λ*_1_ and *k* = *π* for *λ*_2_ = *μ* + *λ*_1_), as a consequence of it the loop always touch the origin of the auxiliary space. Thus the system is in the bulk gapless non-topological state for these two quantum critical lines. There is no gapless excitation for integer topological characterization except at the topological quantum phase transition point, we observe that the loop only touch the origin for that situation.

We show explicitly in the “Method” section that the system has only one bulk gapless excitation for the quantum critical line *λ*_2_ = *μ* + *λ*_1_, whereas the quantum line, *λ*_2_ = *μ* − *λ*_1_, has two bulk gapless edge modes.

Now we discuss the another interpretation based on redefinition of the “ill-define” points. The band gap always vanishes at a specific momentum along a quantum critical line, if we exclude the specific momentum where the band gap vanishes along the quantum critical lines, when calculating the winding number, thus the fractional topological characterization preserve and the edge mode exist. Thus the physical picture and also the difference of integer and fractional topological characterization has explained explicitly in this study.

**Figure 6:**
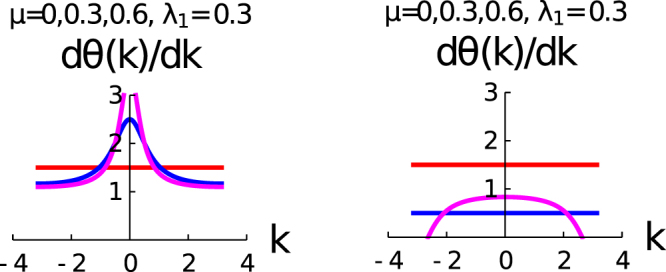
Shows the variation of $(\frac{d\theta (k)}{dk})$ with *k*. Different curves are for different values of *μ*, red (*μ* = 0), blue (*μ* = 0.3), magenta (*μ* = 0.6) respectively. The left panel is for the *λ*_2_ = *μ* + *λ*_1_ and the right panel is for *λ*_2_ = *μ* − *λ*_1_.

In Fig. 6, we present the study of $\frac{d\theta (k)}{dk}$ with *k* for the fractional topological characterization regime of the parameter space. The left panel of the figure is for the *λ*_2_ = *μ* + *λ*_1_ and the right panel of the figure is for *λ*_2_ = *μ* − *λ*_1_. We observe for both the cases *μ* = 0, $\frac{d\theta (k)}{dk}$ is constant and a fraction, as a consequence of it *γ*^(4)^ is also a fractional value of *π*. For the right panel of this figure, we observe two constant lines of $\frac{d\theta (k)}{dk}$ for *μ* = 0,0.3. These two constant lines correspond to two different fractional values of winding number. The behavior of $\frac{d\theta (k)}{dk}$ with *k* for the left panel and right panel of the figures are different for non-zero values of *μ*. The variation of $\frac{d\theta (k)}{dk}$ is symmetric with *k*, but this symmetric behavior of fractional topological characterization is different from the integer topological characterization.

### Exact calculations based results for quantization of geometric phase study

In the previous section, we have presented the results based on *γ* (Equations 18–22). It is not possible to find the exact solution for the entire regime of the parameter space.

Here, we find quite a few exact solutions for our model Hamiltonian in the different regime of parameter space and the related physics is discussed. This exact solutions also show difference between the integer and the fractional topological characterization.

The integrand of the expression for the geometric phase for *γ*^(1)^ and *γ*^(2)^ are in the following form^77^

23 $$\begin{array}{rcl}\int \frac{A+B\,\cos \,x}{a+b\,\cos \,x}dx & = & \frac{B}{b}x+\frac{\mathrm{2(}Ab-aB)}{b\sqrt{{a}^{2}-{b}^{2}}}arc\,\tan (\frac{\sqrt{{a}^{2}-{b}^{2}}\,\tan \,(x\mathrm{/2)}}{a+b})\,({a}^{2}\, > \,{b}^{2})\\  & = & \frac{B}{b}x+\frac{(Ab-aB)}{b\sqrt{{b}^{2}-{a}^{2}}}\,\mathrm{Log}\,(\frac{\sqrt{{b}^{2}-{a}^{2}}\,\tan \,(x\mathrm{/2)}+(a+b)}{\sqrt{{b}^{2}-{a}^{2}}\,\tan \,(x\mathrm{/2)}-(a+b)})\,({b}^{2}\, > \,{a}^{2}\mathrm{).}\end{array}$$

1. Exact solutions for *λ*_1_ ≠ 0 and *λ*_2_ = 0:
  We observe from the analytical expression of *γ*^(1)^ = *π* for *μ* = 0 for all the values of *λ*_1_. We also observe that this transition occurs at *λ*_1_ = *μ* (upper row of Fig. 1), i.e., the *γ*^(1)^ jumps from 0 to *π*. Now we look to find a result from the exact analytical expression for arbitrary *λ*_1_ and *μ*. At first, we present the results for *λ*_1_ ≠ 0 and *λ*_2_ = 0. For this regime of parameter space is (Equation 18):
  $A=2{{{\lambda }}_{1}}^{2},B=-\,2\mu {{\lambda }}_{1}$. $a=4{{\lambda }_{1}}^{2}+4{\mu }^{2},b=-\,8\mu {\lambda }_{1}$. The result which we obtain for indefinite integral by using the first equation of Eq. (23) is the following,
  $${g}_{1}(k,{\lambda }_{1},\mu )=\frac{k}{4}+\frac{1}{\mathrm{2(}{\lambda }_{1}-\mu )}arc\,\tan \,(({\lambda }_{1}+\mu )\,\tan \,(k\mathrm{/2));}\mathop{\mathrm{lim}}\limits_{\delta \to {0}^{+}}{g}_{1}(k,{\lambda }_{1},\mu {)|}_{k=-\,\pi +\delta }^{k=\pi -\delta }=\pi .$$
  We obtained this result for the condition *λ*_1_ > *μ*.
2. Exact solutions for *λ*_2_ ≠ 0 and *λ*_1_ = 0:
  We observe from the analytical expression of *γ*^(2)^ = 2*π* that for *μ* = 0 for the all values of *λ*_2_. Topological quantum phase transition occurs at *λ*_2_ = *μ* (middle row of Fig. 1). Now the question is how to get the results from the exact analytical expression for arbitrary *λ*_2_ and *μ*. For this regime of parameter space (we get it from Equation. 19):
  $A=4{{\lambda }_{2}}^{2},B=-\,4\mu {\lambda }_{2}$. $a=4{{\lambda }_{2}}^{2}+4{\mu }^{2},b=-\,8\mu {\lambda }_{2}$. The result which we obtain for indefinite integral by using the first equation of Equation (23) is the following,
  $${g}_{2}(k,{\lambda }_{2},\mu )=\frac{k}{2}+\frac{1}{({\lambda }_{2}-\mu )}arc\,\tan \,(({\lambda }_{2}+\mu )\,\tan \,(k\mathrm{/2));}\mathop{\mathrm{lim}}\limits_{\delta \to {0}^{+}}{g}_{2}(k,{\lambda }_{2},\mu {)|}_{k=-\,\pi +\delta }^{k=\pi -\delta }\mathrm{=2}\pi .$$
  We obtain this result for the condition *λ*_2_ > *μ*.
3. Exact solutions for *λ*_1_ ≠ 0, *λ*_2_ ≠ 0, and *μ* = 0:
  In this case, the analytical expression for geometric phase become,
  24 $$\gamma ={\int }_{-\pi }^{\pi }\frac{6{\lambda }_{1}{\lambda }_{2}\,\cos \,k+\mathrm{2(}{{\lambda }_{1}}^{2}+2{{\lambda }_{2}}^{2})}{\mathrm{(4}{{\lambda }_{1}}^{2}+4{{\lambda }_{2}}^{2}+8{\lambda }_{1}{\lambda }_{2}\,\cos \,k)}\,{\rm{mod}}\,\mathrm{(2}\pi \mathrm{).}$$
  For this regime of parameter space:
  $A=\mathrm{2(}{{\lambda }_{1}}^{2}+2{{\lambda }_{2}}^{2}),B=6{\lambda }_{1}{\lambda }_{2}$. $a=4{{\lambda }_{1}}^{2}+4{{\lambda }_{2}}^{2},b=8{\lambda }_{1}{\lambda }_{2}$. Now using Equation 23, we get the following results.
  For *λ*_1_ > *λ*_2_:
  $${g}_{3}(k,{\lambda }_{1},{\lambda }_{2})=\frac{1}{4}(3k-2arc\,\tan \,(\frac{({\lambda }_{1}-{\lambda }_{2})\,\tan \,(k\mathrm{/2)}}{{\lambda }_{1}+{\lambda }_{2}}));\mathop{\mathrm{lim}}\limits_{\delta \to {0}^{+}}{g}_{3}(k,{\lambda }_{1},{\lambda }_{2}{)|}_{k=-\,\pi +\delta }^{k=\pi -\delta }=\pi .$$
  For *λ*_2_ > *λ*_1_:
  $${g}_{4}(k,{\lambda }_{1},{\lambda }_{2})=\frac{3k{{\lambda }_{1}}^{2}-3k{{\lambda }_{2}}^{2}+2\sqrt{-{({{\lambda }_{1}}^{2}-{{\lambda }_{2}}^{2})}^{2}}Log[\frac{{L}_{1}}{{L}_{2}}]}{\mathrm{4(}{{\lambda }_{1}}^{2}-{{\lambda }_{2}}^{2})}.;\mathop{lim}\limits_{\delta \to {0}^{+}}{g}_{4}(k,{\lambda }_{1},{\lambda }_{2}{)|}_{k=-\,\pi +\delta }^{k=\pi -\delta }=2\pi .$$
  Here, $${L}_{1}=-\,{({\lambda }_{1}+{\lambda }_{2})}^{2}+\sqrt{-({{\lambda }_{1}}^{2}-{{\lambda }_{2}}^{2})}tan\,(k\mathrm{/2)},$$
  $${L}_{2}=-{({\lambda }_{1}+{\lambda }_{2})}^{2}-\sqrt{-({{\lambda }_{1}}^{2}-{{\lambda }_{2}}^{2})}\,\tan (k/2),$$
  This is one of the most important results of exact solutions. Here we predict the transition of geometric phase from *π* to 2*π*, i.e., the winding number changes from 1 to 2.
4. The exact solution for the quantum critical line *λ*_2_ = − *μ*: The exact solution for *μ* = 0 is ${\gamma }^{(3)}=\frac{1}{2}{\int }_{-\pi }^{\pi }dk=\pi$ for non-zero values of *λ*_1_. We obtain the exact value of *γ*^(3)^ = *π* for *μ* = *λ*_1_. We also observe that there is no transition of *γ*^3^ at *λ*_1_ = *μ*, ${\gamma }^{(3)}=\frac{1}{2}{\int }_{-\pi }^{\pi }dk=\pi$.
5. The exact solution for the quantum critical line (*λ*_2_ = *μ* + *λ*_1_):
  We obtain for *μ* = 0, value of ${\gamma }^{(4)}={\int }_{-\pi }^{\pi }\frac{6{{\lambda }_{1}}^{2}(1+\,\cos \,k)}{8{{\lambda }_{1}}^{2}(1+\,\cos \,k)}=\frac{3\pi }{2}$. We exclude the point *k* = *π*, during the integration. When *μ* = *λ*_1_, ${\gamma }^{(4)}={\int }_{-\pi }^{\pi }\frac{10{\mu }^{2}\,\cos \,k+2(9{\mu }^{2}-4{\mu }^{2}\,\cos \,2k)}{24{\mu }^{2}+8{\mu }^{2}\,\cos \,k-16{\mu }^{2}\,\cos \,2k}=\frac{3\pi }{2}$. Thus, the exact solution also predicts the fractional topological quantization.
6. The exact solution for the quantum critical line *λ*_2_ = *μ* − *λ*_1_:

We obtain for *μ* = 0, $${\gamma }^{(5)}={\int }_{-\pi }^{\pi }\frac{6{{\lambda }_{1}}^{2}(1-\,\cos \,k)}{8{{\lambda }_{1}}^{2}(1-\,\cos \,k)}=\frac{\pi }{2}$$. The exact solution for *λ*_1_ = *μ*, we obtain, $${\gamma }^{(5)}\,=$$$${\int }_{-\pi }^{\pi }\frac{2{\mu }^{2}(1-\,\cos \,k)}{8{\mu }^{2}(1-\,\cos \,k)}\,=$$$$\frac{3\pi }{2}$$. We exclude the point *k* = 0, during the integration. Thus, the exact solution also predict the fractional topological quantization.

These exact solutions for this model Hamiltonian and its variant are absent in the literature.

### An analysis of duality and its relation to the topological characterization

Here, we present the results of duality study of the model Hamiltonian for all regime of parameter space of our interest, including three quantum critical lines, and also its relation with the topological characterization. The other objective of the duality study is to show how the duality transformation manifests in different quantum critical lines.

Duality and the topological state of the system have a relation. The non-zero values of duality operator $< {{\beta }^{z}}_{n+1/2} >$ is to introduce the disorder in the system^78^. This disorderness can be realized in the following form. This operator acting on the perfectly ordered vacuum state (*ψ* = Π_*n*_|↑_*n*_>) introduce a kink in the system, which is a topological excitation.

Here, we introduce the order and disorder operator following the study of duality of the model Hamiltonian explicitly. These operators are defining the sites of the dual lattice, i.e., we define the operator between the nearest-neighbor site and of the original lattice. The analytical relation between the Pauli operators and *β* operators are the following:

25 $${{\beta }_{z}}^{2}=1={{\beta }_{x}}^{2},$$

26 $${\beta }_{z}(n-1/2){\beta }_{z}(n+1/2)={\sigma }_{x}(n).$$

27 $${\beta }_{x}(n+1/2)={\sigma }_{z}(n){\sigma }_{z}(n+1),$$

28 $${\beta }_{z}(n+1/2)={{\rm{\Pi }}}_{j=1}^{n}{\sigma }_{x}(j).$$

29 $${\sigma }_{z}(n)={{\rm{\Pi }}}_{j=0}^{n-1}{\beta }_{x}(j+1/2).$$

Now we write the Hamiltonian in terms of the dual operators. The present Hamiltonian (Equation 3) consists of two parts. At first, we study the duality of the first part of the Hamiltonian, i.e., the quantum Ising model with NN interaction only and then we consider the presence of next-nearest-neighbor interaction.

30 $${H}_{1}=-\,\sum _{n=1}^{N}({\lambda }_{1}{{\sigma }^{z}}_{n}{{\sigma }^{z}}_{n+1}+\mu {{\sigma }^{x}}_{n}).$$

After using all the relations between the order and disorder operators, finally, we obtain the duality transform Hamiltonian as,

31 $${H}_{1}=-\,\sum _{n=1}^{N}(\mu {{\beta }^{z}}_{n-1/2}{{\beta }^{z}}_{n+1/2}+{\lambda }_{1}{{\beta }^{x}}_{n+1/2}).$$

It is clear from the above Hamiltonian that it is duality invariant under the exchange of *μ* and *λ*_1_. Now, we do the duality transformation for the total Hamiltonian (Equation 3),

32 $$H=-\,\sum _{n}[\mu {{\sigma }_{n}}^{x}+{\lambda }_{1}{{\sigma }_{n}}^{z}{{\sigma }_{n+1}}^{z}+{\lambda }_{2}{{\sigma }_{n}}^{x}{{\sigma }_{n-1}}^{z}{{\sigma }_{n+1}}^{z}\mathrm{].}$$

Now, we use the relations between the order and disorder operators to study the duality invariant study of the total Hamiltonian.

$$\begin{array}{rcl}{\sigma }_{n}^{x}{{\sigma }_{n-1}}^{z}{{\sigma }_{n+1}}^{z} & = & {\sigma }_{n-1}^{z}{{\sigma }_{n}}^{x}{{\sigma }_{n+1}}^{z}={\sigma }_{n-1}^{z}{{\sigma }_{n}}^{z}{{\sigma }_{n}}^{z}{{\sigma }_{n}}^{x}{{\sigma }_{n+1}}^{z},\\  &  & -{\sigma }_{n-1}^{z}{{\sigma }_{n}}^{z}{{\sigma }_{n}}^{x}{{\sigma }_{n}}^{z}{{\sigma }_{n+1}}^{z},-{{\beta }_{n-1/2}}^{x}{{\beta }_{n-1/2}}^{z}{{\beta }_{n+1/2}}^{z}{{\beta }_{n+1/2}}^{x},\\  & = & -i{{\beta }^{y}}_{n-1/2}(i){{\beta }^{y}}_{n+1/2}=+{{\beta }_{n-1/2}}^{y}{{\beta }_{n+1/2}}^{y}.\end{array}$$

The final modified Hamiltonian is,

33 $$H=-\,\sum _{n}[\mu {{\beta }^{z}}_{n-1/2}{{\beta }^{z}}_{n+1/2}+{\lambda }_{1}{{\beta }^{x}}_{n+1/2}+{\lambda }_{2}{{\beta }^{y}}_{n-1/2}{{\beta }^{y}}_{n+1/2}].$$

Now we see how the duality transform the Hamiltonian at the quantum critical lines.

(A) For the quantum critical line *λ*_2_ = −*μ*,
  34 $${H}^{(A)}=-\,\sum _{n}[\mu ({{\beta }^{z}}_{n-1/2}{{\beta }^{z}}_{n+1/2}-{{\beta }_{n-1/2}}^{y}{{\beta }_{n+1/2}}^{y})+{\lambda }_{1}{{\beta }^{x}}_{n+1/2}].$$
(B) For the quantum critical line *λ*_2_ = *μ* + *λ*_1_,
  35 $${H}^{(B)}=-\,\sum _{n}[\mu ({{\beta }^{z}}_{n-1/2}{{\beta }^{z}}_{n+1/2}+{{\beta }_{n-1/2}}^{y}{{\beta }_{n+1/2}}^{y})+{\lambda }_{1}({{\beta }^{x}}_{n+1/2}+{{\beta }_{n-1/2}}^{y}{{\beta }_{n+1/2}}^{y})]$$
(C) For the quantum critical line *λ*_2_ = *μ* − *λ*_1_,
  36 $${H}^{(C)}=-\,\sum _{n}[\mu ({{\beta }^{z}}_{n-1/2}{{\beta }^{z}}_{n+1/2}+{{\beta }_{n-1/2}}^{y}{{\beta }_{n+1/2}}^{y})+{\lambda }_{1}({{\beta }^{x}}_{n+1/2}-{{\beta }_{n-1/2}}^{y}{{\beta }_{n+1/2}}^{y})].$$
(D) For the NNN interaction only, *λ*_2_ ≠ 0,*λ*_1_ = 0,

37 $${H}^{(D)}=-\,\sum _{n}[\mu ({{\beta }^{z}}_{n-1/2}{{\beta }^{z}}_{n+1/2}+{{\beta }_{n-1/2}}^{y}{{\beta }_{n+1/2}}^{y})+{\lambda }_{2}{{\beta }_{n-1/2}}^{y}{{\beta }_{n+1/2}}^{y}].$$

We have shown that the Hamiltonian *H*_1_, *H*^(*A*)^, *H*^(*D*)^ show the integer topological characterization, and, we observe that the Hamiltonian, *H*_1_ is only duality invariant. The Hamiltonian *H*^(*B*)^ and *H*^(*C*)^ show the fractional topological characterization and at the same time duality is not preserved. Therefore, the invariance and breakdown of duality is independent whether it is in the integer or fractionally topological characterization, it depends only on the range of the interaction.

To the best of our knowledge, this is the first study in the literature of quantum Ising models to find the relations between the duality for all quantum critical lines and its relation to the topological characterization, i.e., whether the duality depends on the nature of topological characterization.

### Symmetry study of the model Hamiltonian

In this section, we present the result of Time-reversal symmetry ($\hat{{\rm{\Theta }}}$), Charge-conjugation symmetry ($\hat{{\rm{\Xi }}}$), Chiral ($\hat{{\rm{\Pi }}}$) symmetry, Parity symmetry (*P*), CT symmetry, PT symmetry and CPT symmetries of the model Hamiltonian. We do this study of symmetries for three reasons^79–82^: In the first point, we have already discussed *γ* is related *W*, when the model Hamiltonian of the system obeys the anti-unitary particle-hole symmetry, it triggers us to study the other different symmetries of the model Hamiltonian. The second point is that whether the symmetries of the model Hamiltonian manifests in different way for the integer and fractional topological characterization. The third point is that the non-Hermitian Hamiltonian which shows the anomalous edge modes with fractional value of winding number possess the chiral symmetry and PT symmetry. Therefore, it also motivates us to study the symmetry properties of this model Hamiltonian system and how it responses for the fractional and integer topological characterization.

Here, we only present the basic aspects of symmetry and the results of symmetry study. The detailed calculations are relegated to the “Method” section. The BdG Hamiltonian of the present problem is for spinless fermion, and, therefore, we use the spinless format of the symmetry operators. We use the properties of *χ*_*y*_(*k*) and *χ*_*z*_(*k*) during the derivation of these results.

Now we present the results of symmetry studies of this model Hamiltonian. The detail derivations are relegated to the “Method” section.

**Time reversal symmetry operation:**

$${\hat{{\rm{\Theta }}}}^{\dagger }{\hat{H}}_{BdG}(k)\hat{{\rm{\Theta }}}={\hat{K}}^{\dagger }{\hat{H}}_{BdG}(k)\hat{K}\mathrm{.}$$

$${\hat{{\rm{\Theta }}}}^{\dagger }{\hat{H}}_{BdG}(k)\hat{{\rm{\Theta }}}=\hat{K}[\begin{array}{cc}{\chi }_{z}(k) & i{\chi }_{y}(k)\\ -i{\chi }_{y}(k) & -{\chi }_{z}(k)\end{array}]\hat{K}={H}_{BdG}.$$

Thus, the model Hamiltonian is invariant under time reversal symmetry.

**Charge-conjugation symmetry:**

Charge-conjugation symmetry operator is $\hat{{\rm{\Xi }}}$.

$${\hat{{\rm{\Xi }}}}^{\dagger }\hat{H}(k)\hat{{\rm{\Xi }}}={({\sigma }_{x}\hat{K})}^{\dagger }\hat{H}(k)({\sigma }_{x}\hat{K})={\hat{K}}^{\dagger }{\sigma }_{x}\hat{H}(k){\sigma }_{x}\hat{K}=-\,H(K).$$

Thus, the model Hamiltonian is invariant under charge-conjugation symmetry.

**Chiral symmetry:**

Chiral symmetry operator is given by, $\hat{{\rm{\Pi }}}$.

$${\hat{{\rm{\Pi }}}}^{\dagger }{\hat{H}}_{BdG}(k)\hat{{\rm{\Pi }}}={\sigma }_{x}{\hat{H}}_{BdG}(k){\sigma }_{x}=-\,{H}_{BdG}.$$

Thus, the model Hamiltonian is invariant under chiral symmetry.

**Parity symmetry** *PH*_*BdG*_(*k*)*P*^−1^ = *σ*_*z*_*H*_*BdG*_(*k*)*σ*_*z*_ = *H*_*BdG*_(−*k*)

Thus, the Hamiltonian obeys parity symmetry.

**CP symmetry** *CPH*_*BdG*_(*k*)(*CP*)^−1^ = *σ*_*x*_*Kσ*_*z*_*H*_*BdG*_(*k*)*σ*_*z*_*K*^−1^*σ*_*x*_ = −*H*_*BdG*_(−*k*).

Thus, the Hamiltonian obeys CP symmetry.

**PT symmetry:**

$$PT{H}_{BdG}(k){(PT)}^{-1}={{\sigma }}_{z}K{H}_{BdG}(k){K}^{-1}{{\sigma }}_{z}\ne {H}_{BdG}(k).$$

Thus, the Hamiltonian does not obeys PT symmetry.

**CT symmetry:**

$$CT{H}_{BdG}(k){(CT)}^{-1}={\sigma }_{x}{H}_{BdG}(k){\sigma }_{x}=-\,{H}_{BdG}.$$

Thus, the Hamiltonian obeys CT symmetry.

**CPT symmetry:**

$$\alpha {H}_{BdG}(k){\alpha }^{-1}={\sigma }_{x}{\sigma }_{z}K{H}_{BdG}(k){K}^{-1}{\sigma }_{z}{\sigma }_{x}\ne -\,{H}_{BdG}(k).$$

Thus, the Hamiltonian does not obey CPT symmetry.

Now we compare the relations between the duality and symmetry: For the NN coupling the duality is invariant and the results obtained from the Zak phase study is also physically consistent. In the presence of NNN or further long range interaction duality breaks down for this model Hamiltonian but the behaviour of the symmetry, whether it is preserved (time reversal, chiral, parity CP and CT) or breakdown (PT and CPT), is the same for NN, NNN and any long range interaction. Therefore, the duality and symmetry behaves differently depending on the range of interaction.

### Effect of further long range interaction

Here, we have studied the interaction up to NNN interactions. One can generalize these results for further long range interaction. Suppose one consider the third NN interaction, the integer winding number will be 0, 1, 2, 3 and the fractional winding number will be 5/2, 3/2, 1/2. Similarly, one can generalize it for further long range interaction with maintain the sequence of NN and NNN interactions. The only thing is that the properties of duality and symmetry will be the same as we obtain from the study of NNN interactions, i.e., the NNN interaction is sufficient to characterize the complete nature of duality and symmetry for this model Hamiltonian system.

## Discussion

We have presented the quantization of geometric phase for both integer and fractionalize topological characterization for this system along with the physical explanations. We have shown that all the quantum critical lines are not topologically equivalent. We have also presented the exact solutions based results for the quantization of geometric phase. The model Hamiltonian possesses different symmetries and the symmetry properties are the same for all quantum critical lines. One of, the most important conclusion is that Zak phase is not the proper physical quantity for the characterization of topological state in the presence of long range interactions. One can generalize this result for further long range interaction. We have shown explicitly relation between the duality, symmetry and topological characterization. Our work provides a new perspective of topological quantization.

## Method

### Detailed derivation of quantum critical line

We start with the Hamiltonian *H*_2_ (Equation 5),

38 $$\begin{array}{rcl}{H}_{2} & = & -2\sum _{k > 0}(-\,{\mu }+{{\lambda }}_{1}\,\cos \,k+{\lambda }_{2}\,\cos \,2k)({{c}_{k}}^{\dagger }{c}_{k}+{{c}_{-k}}^{\dagger }{c}_{-k})\\  &  & +2i\sum _{k > 0}({\lambda }_{1}\,\sin \,k+{\lambda }_{2}\,\sin \,2k)({{c}_{k}}^{\dagger }{{c}_{-k}}^{\dagger }+{c}_{k}{c}_{-k}).\end{array}$$

Now our main task is to express the Hamiltonian in the diagonalized form. We follow the Bogoliubov transformation ${\eta }_{k}={\alpha }_{k}{c}_{k}+i{\beta }_{k}{{c}_{-k}}^{\dagger }$ and ${\eta }_{-k}={\alpha }_{k}{c}_{-k}-i{\beta }_{k}{{c}_{k}}^{\dagger }$, *k* > 0.

The operator *η*_*k*_ and ${{{\eta }}_{k}}^{\dagger }$ are the fermionic operators. We use the following relations,

$\{{\eta }_{k},{{\eta }_{p}}^{\dagger }\}={\delta }_{k,p}$, {*η*_*k*_, *η*_*p*_} = 0, $\{{{\eta }_{k}}^{\dagger },{{\eta }_{p}}^{\dagger }\}=0$. This relation implies ${{\alpha }_{k}}^{2}+{{\beta }_{k}}^{2}=1$.

One can also revert the relation between *c*_*k*_ and *η*_*k*_. We also parameterize *α*_*k*_ = cos *θ*_*k*_ and *β*_*k*_ = sin *θ*_*k*_. One can express the transformed Hamiltonian in two parts

39 $${H}_{2}={H}_{2A}+{H}_{2B}$$

40 $$\begin{array}{rcl}{H}_{2A} & = & \sum _{k\mathrm{ > 0}}[\,-\,\mathrm{2(}\,-\,\mu +{\lambda }_{1}\,\cos \,k+{\lambda }_{2}\,\cos \,2k)({{\alpha }_{k}}^{2}-{{\beta }_{k}}^{2})\\  &  & +\mathrm{4(}{\lambda }_{1}\,\sin \,k+{\lambda }_{2}\,\sin \,2k){\alpha }_{k}{\beta }_{k}]({{\eta }_{k}}^{\dagger }{\eta }_{k}{{\eta }_{-k}}^{\dagger }{\eta }_{-k}\mathrm{).}\end{array}$$

41 $$\begin{array}{rcl}{H}_{2B} & = & \sum _{k\mathrm{ > 0}}\mathrm{[4}i(\,-\,\mu +{\lambda }_{1}\,\cos \,k+{\lambda }_{2}\,\cos \,2k){\alpha }_{k}{\beta }_{k}\\  &  & +2i({\lambda }_{1}\,\sin \,k+{\lambda }_{2}\,\sin \,2k)({{\alpha }_{k}}^{2}-{{\beta }_{k}}^{2})]({{\eta }_{k}}^{\dagger }{{\eta }_{-k}}^{\dagger }{\eta }_{k}{\eta }_{-k}\mathrm{).}\end{array}$$

To express this Hamiltonian in the diagonal form, we find the following relation

$$\mathrm{4(}\,-\,\mu +{\lambda }_{1}\,\cos \,k+{\lambda }_{2}\,\cos \,2k){\alpha }_{k}{\beta }_{k}+\mathrm{(2}i{\lambda }_{1}\,\sin \,k+2i{\lambda }_{2}\,\sin \,2k)({{\alpha }_{k}}^{2}-{{\beta }_{k}}^{2})=0.$$

2*α*_*k*_*β*_*k*_ = sin2*θ*_*k*_, ${{\alpha }_{k}}^{2}-{{{\beta }}_{k}}^{2}=\,\cos \,2{{\theta }}_{k}$. Finally this gives the condition,

42 $$\tan \,2{\theta }_{k}=-\,\frac{{\lambda }_{1}\,\sin \,k+{\lambda }_{2}\,\sin \,2k}{{\lambda }_{1}\,\cos \,k+{\lambda }_{2}\,\cos \,2k-\mu }\mathrm{.}$$

Finally, we write the Hamiltonian as,

43 $${H}_{2}=\sum _{k}{E}_{k}({\lambda }_{1},{\lambda }_{2}){{\eta }_{k}}^{\dagger }{\eta }_{k}$$

44 $$E(k,{\lambda }_{1},{\lambda }_{2})=\sqrt{{{\chi }_{z}}^{2}(k)+{{\chi }_{y}}^{2}(k)}$$

Now, we derive the analytical expressions for quantum critical lines based on the energy dispersion of *E*_*k*_(*λ*_1_, *λ*_2_). The analytical expressions for *χ*_*z*_(*k*) and *χ*_*y*_(*k*) are given in Equations 9 and 10 respectively. The energy gap vanishes at this quantum critical lines, i.e., |*E*(*k*, *λ*_1_, *λ*_2_)| = 0.

First, we consider for *k* = 0 case.

|*E*(0, *λ*_1_, *λ*_2_)| = 0, from this condition, we find the analytical expression for quantum critical line is *λ*_2_ = *μ* − *λ*_1_.

Second, we consider for *k* = *π* case.

|*E*(*π*, *λ*_1_, *λ*_2_)| = 0, from this condition, we find the analytical expression for quantum critical line is *λ*_2_ = *μ* + *λ*_1_.

Third, we consider for $k={\cos }^{-1}(-\,\frac{{\lambda }_{1}}{2{\lambda }_{2}})$ case.

$$|E({\cos }^{-1}(-\frac{{{\lambda }}_{1}}{2{{\lambda }}_{2}}),{{\lambda }}_{1},{{\lambda }}_{2})|=0,$$

From this condition, we find the analytical expression for quantum critical line is *λ*_2_ = −*μ*.

**A detailed analysis to search the parametric relations at the topological quantum phase transition point**

45 $${\chi }_{y}(k)=2{\lambda }_{1}\,\sin \,k+2{\lambda }_{2}\,\sin \,2k,$$

46 $${\chi }_{z}(k)=-\,2{\lambda }_{1}\,\cos \,k-2{\lambda }_{2}\,\cos \,2k+2\mu ,$$

The analytical expression for winding angle is,

$${\theta }_{k}=ta{n}^{-1}(\frac{{\chi }_{y}(k)}{{\chi }_{z}(k)}).$$

At first we discuss the parametric relations for the quantum critical lines

(1). *λ*_2_ = *μ* + *λ*_1_ and (2). *λ*_2_ = *μ* − *λ*_1_.

1. The parametric relations for the quantum critical line *λ*_2_ = *μ* + *λ*_1_ become
  47 $${\chi }_{y}(k)=2{\lambda }_{1}\,\sin \,k+\mathrm{2(}\mu +{\lambda }_{1})\,\sin \,2k,$$
  48 $${\chi }_{z}(k)=-\,2{\lambda }_{1}\,\cos \,k-2(\mu +{\lambda }_{1})\,\cos \,2k+2\mu ,$$
  Now we find the values of *k*s where *χ*_*y*_(*k*) = 0 = *χ*_*z*_(*k*) and we find the parametric relations at the topological quantum phase transition point. From the analysis of *χ*_*y*_(*k*), we get that *χ*_*y*_(*k*) = 0 for the two values of *k*, that are respectively *k*_1_ = 0 and ${k}_{2}={\cos }^{-1}(-\frac{{\lambda }_{1}}{2{\lambda }_{1}+\mu })$. The corresponding values of *χ*_*z*_ are *−*4*λ*_1_ and 2*(λ*_1_ + 2*μ*) respectively. Here *λ*_1_ and *μ* both are positive, therefore it is clear that there is no situation for this quantum critical line with a parametric relation that satisfy $(\frac{{\chi }_{y}(k)}{{\chi }_{z}(k)})=0/0$.
  Now we discuss the third point, i.e., *k*_3_ = *π*, which gives *χ*_*y*_ = *χ*_*z*_ = 0. So there is a gap closing point at *k*_3_ = *π* for this quantum critical line and the system is always in gapless excitation and does not satisfy the gapped topological excitation state, although we get the fractional values of winding number. This excitation is the bulk gapless excitation, there is no edge excitations, which occurs in the integer topological characterization. We only obtain the fractional topological characterization with edge excitations, If we redefine the ill define point for this quantum critical line.
2. Analysis for the other quantum critical line *λ*_2_ = *μ* − *λ*_1_. From the analysis of *χ*_*y*_(*k*), we get that *χ*_*y*_(*k*) = 0 for the two values of *k*, that are respectively *k*_1_ = 0 and ${k}_{2}={\cos }^{-1}(-\frac{{\lambda }_{1}}{2{\lambda }_{1}-\mu })$. For *k*_1_ = 0, *χ*_*y*_(*k*_1_) = 0 *χ*_*z*_(*k*_1_) = 0, and for *k*_2_, *χ*_*z*_(*k*_2_) = 2(*λ*_1_ − 2*μ*). So for *k* = 0, we get this condition $(\frac{{\chi }_{y}(k)}{{\chi }_{z}(k)})=0/0$ satisfy and the system is in always gapless phase.
  It is clear from the analysis for *k*_2_ this quantum critical line satisfy the condition $(\frac{{\chi }_{y}(k)}{{\chi }_{z}(k)})=0/0$ for *μ* = *λ*_1_/2. This is the parametric relation where the another gapless phase occurs. This excitation is the bulk excitation which differ from the edge excitation of integer topological characterization. We only obtain the fractional topological characterization with edge excitations, If we redefine the ill define point for this quantum critical line.
  The numbers of bulk gapless edge modes are different for these two quantum critical lines. It is one for *λ*_2_ = *μ* + *λ*_1_ and two for *λ*_2_ = *μ* − *λ*_1_.
  Now we discuss the situation of parametric relation for integer topological characterization:
3. *λ*_1_ ≠ 0, *λ*_2_ = 0, the gap vanishes only at *k* = 0. Therefore it is clear from the above analysis for *k* this quantum critical line satisfy the condition $(\frac{{\chi }_{y}(k)}{{\chi }_{z}(k)})=0/0$. This occurs for *χ*_*z*_(*k*) = 2(*λ*_1_ − *μ*), i.e., the condition satisfy when *λ*_1_ = *μ*. For this situation, the system has gapless edge mode except when *λ*_1_ = *μ*.
4. *λ*_1_ = 0, *λ*_2_ ≠ 0, the gap vanishes only at *k* = 0. Therefore, it is clear from the above analysis for *k* this quantum critical line satisfy the condition $(\frac{{\chi }_{y}(k)}{{\chi }_{z}(k)})=0/0$. This occurs for *χ*_*z*_(*k*) = 2(*λ*_2_ − *μ*), i.e., the condition satisfy when *λ*_2_ = *μ*. We also observe this transition in Fig. 1 (1st and 2nd row). For this situation, the system has gapless edge mode except at *λ*_2_ = *μ*.
5. The other quantum critical line is the *λ*_2_ = −*μ*, here we have not found any parametric relation to show the topological quantum phase transition. For this situation, the system is in the integer topological characterization for the gapped phase (*λ*_1_ > 2*λ*_2_) and in the fractional topological characterization for gapless phase (*λ*_1_ < 2*λ*_2_). This result has shown explicitly in Fig. 2.

### Detailed derivation of symmetries for this model Hamiltonian

At first we introduce the basic concept of the different symmetries very briefly and then the detail derivation of the symmetry operations^73–76^ for this model Hamiltonian.

#### Time-reversal symmetry

This symmetry operation can be represented by an operator $\hat{{\rm{\Theta }}}$. This operator reverses the arrow of time, i.e., *t* → −*t*. The condition for the time-reversal invariant is,

${\hat{{\rm{\Theta }}}}^{\dagger }\hat{H}(k)\hat{{\rm{\Theta }}}=\hat{H}(\,-\,k)$; $[\hat{{\rm{\Theta }}},\hat{H}(k)]=0$. In general, $\hat{{\rm{\Theta }}}$ can be written as, $\hat{{\rm{\Theta }}}=\hat{U}\hat{K},$ where $\hat{U}$ is unitary operator and $\hat{K}$ is complex conjugate operator. The BdG Hamiltonian of the present problem is for spinless fermion, and therefore, $\hat{{\rm{\Theta }}}=\hat{K}$.

#### Charge conjugation symmetry

In this symmetry operation is replacing particle by its antiparticle. $C{{\rm{\Psi }}}_{(p)}={{\rm{\Psi }}}_{(\bar{p})}$ when particles are their own antiparticles and then we have a charge conjugation quantum number *η*^*c*^. *C*Ψ_(*p*)_ = *η*^*c*^Ψ_(*p*)_, *η*^*c*^ is usually taken to be unity.

A generalization of charge-conjugation symmetry with operator, $\hat{{\rm{\Xi }}}$, satisfy the following relation:

$${\hat{{\rm{\Xi }}}}^{\dagger }\hat{H}(k)\hat{{\rm{\Xi }}}={({\sigma }_{x}\hat{K})}^{\dagger }\hat{H}(k)({\sigma }_{x}\hat{K})={\hat{K}}^{\dagger }{\sigma }_{x}\hat{H}(k){\sigma }_{x}\hat{K}.$$

#### Chiral symmetry

A new symmetry can be defined which is called chiral symmetry. It is basically the product of time reversal operator $\hat{\Theta }$ and charge conjugation operator C.

$${\hat{{\rm{\Pi }}}}^{\dagger }\hat{H}(\hat{P},\hat{r})\hat{{\rm{\Pi }}}=-\hat{H}(\hat{P},\hat{r}).$$

#### Parity symmetry

This symmetry is discrete unitary symmetry of nature. Parity symmetry is a transformation of all spatial coordinate axes, i.e., $\hat{P}$: r → −r, where $\hat{P}$ is parity operator. Thus, parity transformation flips the sign of all spatial axes and also the momentum. Square of parity operator equals to identity (${\hat{P}}^{2}\mathrm{=1}$), and thus it has eigenvalues ±1. $\hat{P}=1$ and $\hat{P}=-\,1$ corresponds to even and odd parity states respectively. If a Hamiltonian is invariant under parity it obeys $\hat{P}H(\overrightarrow{p}){\hat{P}}^{-1}=H(-\,\overrightarrow{p})\,;\,[\hat{P},H]=0.$ Parity operator($\hat{P}$) can be defined in the matrix form as, $\hat{P}={\sigma }_{z}$, where *σ*_*z*_ is one among the Pauli matrices.

#### CP symmetry

This symmetry is the combination of charge conjugation and parity symmetry. CP symmetry is a transformation of particles into antiparticles with the inversion of spatial coordinates. From the definitions of the charge conjugation and parity symmetries, one can write, *CPH*(*k*)(*CP*)^−1^ = −*H*(−*k*), where CP symmetry operator can be given by *CP* = *σ*_*x*_*Kσ*_*z*_.

#### PT symmetry

This symmetry is a combination of parity and time-reversal symmetries. PT symmetry is transformation of space coordinates along with time. In other words, the reflection of space and time coordinates, i.e., *PT*: (*x*, *y*, *z*, *t*) → (−*x*, −*y*, −*z*, −*t*). For a Hamiltonian which is invariant under PT symmetry we can write,

49 $$PTH(p)(PT{)}^{-1}=H(\,-\,\bar{p});\,[PT,H]=0.$$

Since time reversal flips the sign of the spin, we have PT symmetry operator for spinless systems as *PT* = *σ*_*z*_*K*.

#### CT symmetry

This symmetry is a combination of charge conjugation and time-reversal symmetry. It is a transformation of particles into antiparticles with inversion of time. For a Hamiltonian which is invariant under CT symmetry must obey this relation, *CTH*(*p*)(*CT*)^−1^ = −*H*(*p*); {*CT*,*H*} = 0. One can write the CT operator for spinless systems as *CT* = *σ*_*x*_*KK* = *σ*_*x*_.

#### CPT symmetry

This symmetry is combination of charge conjugation, parity and time-reversal symmetry. It is a transformation of particles into antiparticle with inversion of all space and time coordinates. We denote CPT symmetry operator as *α*. Hamiltonian which is invariant under CPT symmetry must obey the relation, *αH*(*k*)*α*^−1^ = −*H*(*k*); {*α*, *H*} = 0, where the CPT operator is given by *α* = −*iCPT*. For spinless systems, one can compute the operator as $${\alpha }=-\,iCPT=-\,i{\sigma }_{x}K{\sigma }_{z}K=-\,i{\sigma }_{x}{\sigma }_{z}=(\begin{array}{cc}0 & i\\ -i & 0\end{array})$$

**Time-reversal symmetry**

Time-reversal symmetry operation is $\hat{{\rm{\Theta }}}$.

$${\hat{{\rm{\Theta }}}}^{\dagger }{\hat{H}}_{BdG}(k)\hat{{\rm{\Theta }}}={\hat{K}}^{\dagger }{\hat{H}}_{BdG}(k)\hat{K}\mathrm{.}\,{\hat{{\rm{\Theta }}}}^{\dagger }{\hat{H}}_{BdG}(k)\hat{{\rm{\Theta }}}=\hat{K}[\begin{array}{cc}{\chi }_{z}(k) & i{\chi }_{y}(k)\\ -\,i{\chi }_{y}(k) & -\,{\chi }_{z}(k)\end{array}]\hat{K}\mathrm{.}$$

$$\begin{array}{rcl}{\chi }_{z}(k) & = & -2{\lambda }_{1}\,\cos \,k-2{\lambda }_{2}\,\cos \,2k+2\mu ,{\chi }_{y}(k)=2{\lambda }_{1}\,\sin \,k+2{\lambda }_{2}\,\sin \,2k,{\chi }_{z}(k)\\  & = & {\chi }_{z}(\,-\,k)\,{\rm{and}}\,{\chi }_{y}(k)=-\,{\chi }_{y}(\,-\,k\mathrm{).}\end{array}$$

$${\hat{{\rm{\Theta }}}}^{\dagger }{\hat{H}}_{BdG}(k)\hat{{\rm{\Theta }}}=[\begin{array}{cc}{\chi }_{z}(\,-\,k) & -i{\chi }_{y}(\,-\,k)\\ i{\chi }_{y}(\,-\,k) & -{\chi }_{z}(\,-\,k)\end{array}]=H(k).$$

It is clear from the above expression that the model Hamiltonian obeys time reversal symmetry. During this derivation, we use the properties of *χ*_*z*_(*k*) = *χ*_*z*_(−*k*) and *χ*_*y*_(*k*) = −*χ*_*y*_(−*k*).

**Charge-conjugation symmetry**

This symmetry operator is $\hat{{\rm{\Xi }}}$.

$$\begin{array}{rcl}{\hat{{\rm{\Xi }}}}^{\dagger }\hat{H}(k)\hat{{\rm{\Xi }}} & = & {({\sigma }_{x}\hat{K})}^{\dagger }\hat{H}(k)({\sigma }_{x}\hat{K})={\hat{K}}^{\dagger }{\sigma }_{x}\hat{H}(k){\sigma }_{x}\hat{K}\\ {\hat{{\rm{\Xi }}}}^{\dagger }\hat{H}(k)\hat{{\rm{\Xi }}} & = & {\hat{K}}^{\dagger }(\begin{array}{cc}0 & 1\\ 1 & 0\end{array})(\begin{array}{cc}{\chi }_{z}(k) & i{\chi }_{y}(k)\\ -i{\chi }_{y}(k) & -\chi (k)\end{array})(\begin{array}{cc}0 & 1\\ 1 & 0\end{array})\hat{K}.\\ {\hat{{\rm{\Xi }}}}^{\dagger }\hat{H}(k)\hat{{\rm{\Xi }}} & = & \hat{K}(\begin{array}{cc}-{\chi }_{z}(k) & -i{\chi }_{y}(k)\\ i{\chi }_{y}(k) & {\chi }_{z}(k)\end{array})\hat{K}=(\begin{array}{cc}-{\chi }_{z}(k) & -i{\chi }_{y}(k)\\ i{\chi }_{y}(k) & {\chi }_{z}(k)\end{array})=-\hat{H}(k)\end{array}$$

Thus, the Hamiltonian obeys charge-conjugation symmetry.

**Chiral symmetry**

This symmetry operator is given by, $\hat{{\rm{\Pi }}}$.

$$\begin{array}{rcl}{\hat{{\rm{\Pi }}}}^{\dagger }{\hat{H}}_{BdG}(k)\hat{{\rm{\Pi }}} & = & {\sigma }_{x}{\hat{H}}_{BdG}(k){\sigma }_{x}\\ {\hat{{\rm{\Pi }}}}^{\dagger }\hat{H}(k)\hat{{\rm{\Pi }}} & = & (\begin{array}{cc}0 & 1\\ 1 & 0\end{array})(\begin{array}{cc}{\chi }_{z}(k) & i{\chi }_{y}(k)\\ -i{\chi }_{y}(k) & -{\chi }_{z}(k)\end{array})(\begin{array}{cc}0 & 1\\ 1 & 0\end{array}).\\ {\hat{{\rm{\Pi }}}}^{\dagger }\hat{H}(k)\hat{{\rm{\Pi }}} & = & (\begin{array}{cc}-i{\chi }_{y}(k) & -{\chi }_{z}(k)\\ {\chi }_{z}(k) & i{\chi }_{y}(k)\end{array})(\begin{array}{cc}0 & 1\\ 1 & 0\end{array})=-(\begin{array}{cc}{\chi }_{z}(k) & i{\chi }_{y}(k)\\ -i{\chi }_{y}(k) & {\chi }_{z}(k)\end{array}).\end{array}$$

${\hat{{\rm{\Pi }}}}^{\dagger }\hat{H}(k)\hat{{\rm{\Pi }}}=-\,\hat{H}(k).$ Thus, the Hamiltonian also obeys chiral symmetry.

**Parity symmetry**

50 $$\begin{array}{rcl}PH(k){P}^{-1} & = & {\sigma }_{z}H(k){\sigma }_{z}\\  & = & (\begin{array}{cc}1 & 0\\ 0 & -1\end{array})(\begin{array}{cc}{\chi }_{z}(k) & i{\chi }_{y}(k)\\ -i{\chi }_{y}(k) & {\chi }_{z}(k)\end{array})(\begin{array}{cc}1 & 0\\ 0 & -1\end{array})\\  & = & (\begin{array}{cc}{\chi }_{z}(k) & i{\chi }_{y}(k)\\ i{\chi }_{y}(k) & {\chi }_{z}(k)\end{array})(\begin{array}{cc}1 & 0\\ 0 & -1\end{array})\\  & = & (\begin{array}{cc}{\chi }_{z}(k) & -i{\chi }_{y}(k)\\ i{\chi }_{y}(k) & -{\chi }_{z}(k)\end{array})=-\,H(\,-\,k)\end{array}$$

Thus, the Hamiltonian obeys parity symmetry.

**CP symmetry**

51 $$\begin{array}{rcl}CPH(k){(CP)}^{-1} & = & {\sigma }_{x}K{\sigma }_{z}H(k){\sigma }_{z}{K}^{-1}{\sigma }_{x}\\  & = & {\sigma }_{x}K(\begin{array}{cc}1 & 0\\ 0 & -1\end{array})(\begin{array}{cc}{\chi }_{z}(k) & i{\chi }_{y}(k)\\ -i{\chi }_{y}(k) & -{\chi }_{z}(k)\end{array})(\begin{array}{cc}1 & 0\\ 0 & -1\end{array}){K}^{-1}{\sigma }_{x}\\  & = & {\sigma }_{x}K(\begin{array}{cc}{\chi }_{z}(k) & -i{\chi }_{y}(k)\\ i{\chi }_{y}(k) & -{\chi }_{z}(k)\end{array}){K}^{-1}{\sigma }_{x}\\  & = & (\begin{array}{cc}0 & 1\\ 1 & 0\end{array})(\begin{array}{cc}{\chi }_{z}(k) & -i{\chi }_{y}(k)\\ i{\chi }_{y}(k) & -{\chi }_{z}(k)\end{array})(\begin{array}{cc}0 & 1\\ 1 & 0\end{array})\\  & = & (\begin{array}{cc}-{\chi }_{z}(k) & i{\chi }_{y}(k)\\ -i{\chi }_{y}(k) & {\chi }_{z}(k)\end{array})=-\,H(\,-\,k)\end{array}$$

Thus, the Hamiltonian obeys CP symmetry.

**PT symmetry**

52 $$\begin{array}{rcl}PTH(k){(PT)}^{-1} & = & {\sigma }_{z}KH(k){K}^{-1}{\sigma }_{z}\\  & = & {\sigma }_{z}H(k){\sigma }_{z}\\  & = & (\begin{array}{cc}1 & 0\\ 0 & -1\end{array})(\begin{array}{cc}{\chi }_{z}(k) & i{\chi }_{y}(k)\\ -i{\chi }_{y}(k) & -{\chi }_{z}(k)\end{array})(\begin{array}{cc}1 & 0\\ 0 & -1\end{array})\\  & = & (\begin{array}{cc}{\chi }_{z}(k) & -i{\chi }_{y}(k)\\ i{\chi }_{y}(k) & -{\chi }_{z}(k)\end{array})\ne H(k)\end{array}$$

Thus the Hamiltonian does not obeys PT symmetry.

**CT symmetry**

53 $$\,\begin{array}{rcl}CTH(k){(CT)}^{-1} & = & {\sigma }_{x}H(k){\sigma }_{x}\\  & = & (\begin{array}{cc}0 & 1\\ 1 & 0\end{array})(\begin{array}{cc}{\chi }_{z}(k) & i{\chi }_{y}(k)\\ -i{\chi }_{y}(k) & {\chi }_{z}(k)\end{array})(\begin{array}{cc}0 & 1\\ 1 & 0\end{array})\\  & = & (\begin{array}{cc}-i{\chi }_{y}(k) & -{\chi }_{z}(k)\\ {\chi }_{z}(k) & i{\chi }_{y}(k)\end{array})(\begin{array}{cc}0 & 1\\ 1 & 0\end{array})\\  & = & (\begin{array}{cc}-{\chi }_{z}(k) & -i{\chi }_{y}(k)\\ i{\chi }_{y}(k) & {\chi }_{z}(k)\end{array})=-H(k)\end{array}$$

Thus, the Hamiltonian obey the CT symmetry.

**CPT symmetry**

54 $$\begin{array}{rcl}\alpha H(k){\alpha }^{-1} & = & {\sigma }_{x}{\sigma }_{z}KH(k){K}^{-1}{\sigma }_{z}{\sigma }_{x}\\  & = & (\begin{array}{cc}0 & 1\\ -1 & 0\end{array})(\begin{array}{cc}{\chi }_{z}(k) & i{\chi }_{y}(k)\\ -i{\chi }_{y}(k) & -{\chi }_{z}(k)\end{array})(\begin{array}{cc}0 & -1\\ 1 & 0\end{array})\\  & = & (\begin{array}{cc}-{\chi }_{z}(k) & i{\chi }_{y}(k)\\ -i{\chi }_{y}(k) & {\chi }_{z}(k)\end{array})\ne -H(k)\end{array}$$

Thus, the Hamiltonian does not obey the CPT symmetry.
